# Testosterone therapy for cognition in menopause: A systematic review and meta-analysis

**DOI:** 10.1530/RAF-26-0080

**Published:** 2026-10-30

**Authors:** Alexandra Faulkner, Roopal Desai, Nicky Keay, Shahara Aziz Ellahi, Aimee Spector

**Affiliations:** ^1^ Department of Clinical, Educational & Health Psychology, University College London, London, United Kingdom; ^2^ Division of Medicine, University College London, London, United Kingdom

**Keywords:** menopause, testosterone, menopause-replacement-therapy, cognition

## Abstract

**Abstract:**

The objective of this review was to explore the effect of testosterone therapy on objective and subjective cognition during the menopause transition and postmenopause through a systematic review and meta-analysis. A systematic search was conducted in PsycINFO, MEDLINE, Embase, Web of Science and the Cochrane Library from database inception to March 2025 using predefined eligibility criteria. A total of 3,880 records were identified. After screening, five studies were eligible for inclusion. Three studies including 244 postmenopausal participants were included in a random-effects meta-analysis to estimate pooled effects. Three cognitive domains were covered: executive function, verbal learning and memory and visuospatial learning and memory. Studies excluded from the meta-analysis were reported narratively. The meta-analysis found no statistically significant pooled effects of testosterone therapy across the cognitive domains analysed, with effect sizes mostly close to zero. No studies explored the effect of testosterone therapy on subjective cognition or during perimenopause. The findings should be interpreted as insufficient to establish an effect rather than as evidence of no effect, given the small number of available studies and their limited sample sizes. Further adequately powered trials are needed to investigate the effects of testosterone therapy on cognition, particularly during the menopause transition, the period in which cognitive problems appear to be most pronounced.

**Lay summary:**

Menopause is a stage of life that can affect thinking and memory, often colloquially referred to as ‘brain fog’. Brain fog can affect daily life and relationships. Hormone treatments are often used to manage symptoms, but it is still unclear how well they work. Testosterone is sometimes prescribed for low sex drive, but its effects on thinking and memory remain unclear. This study reviews the existing evidence. Five studies were identified. These studies examined different areas of thinking and memory, including attention, planning, decision-making and learning. We found no clear evidence that testosterone affects thinking and memory. However, only a few small studies were available, so the results need to be interpreted carefully. This research highlights a knowledge gap. Further studies are needed on testosterone to guide effective treatment and support women to make informed decisions about their health.

## Introduction

Menopause transition (MT) and postmenopause are life stages in a woman’s midlife marked by hormonal changes. The term ‘women’ is used in this paper to maintain consistency and clarity in relation to sex-based aspects of health (Gribble *et al.* 2022). The authors acknowledge that different terms may be appropriate in different contexts and for different purposes. MT refers to the period between the onset of menstrual changes (perimenopause) and 12 consecutive months without a menstrual period (menopause). Symptoms during these stages may include vasomotor symptoms (e.g. hot flushes and night sweats) (Gold *et al.* 2006), sleep difficulties (Kravitz *et al.* 2003), as well as depression (Bromberger *et al.* 2007) and anxiety (Bromberger *et al.* 2013). With increasing life expectancy, many women will spend a third of their lives in the postmenopausal stage (Schmidt *et al.* 2025).

### Cognition in menopause transition and postmenopause

MT and postmenopause have also been associated with cognitive complaints, colloquially described as ‘brain fog’. This includes difficulties recalling words and numbers, misplacing items, trouble concentrating and forgetfulness (Sullivan & Woods 2001). These may be subjective (women noticing or worrying about changes in their cognition) or objective (changes observed on neuropsychological tests). Subjective complaints are frequently reported in research (Schaafsma *et al.* 2010, Bazeley *et al.* 2022), whereas the research is less unanimous for objective complaints and shows variation across cognitive domains (Unkenstein *et al.* 2016, Kilpi *et al.* 2020, Maki & Weber 2021, Naysmith *et al.* 2026). It has been suggested that subjective cognitive symptoms are weakly associated with objective performance, but moderately related to psychological symptoms (Naysmith *et al.* 2026).

### Hormonal changes in menopause transition and postmenopause

Changes in sex hormones characterise menopause. Testosterone levels do not acutely change during menopause but instead begin to decline from a woman’s mid-20s to early 30s (Davis & Wahlin-Jacobsen 2015, Australian Menopause Society 2019). Sex hormone changes during this period are not uniform and are influenced by factors such as race, ethnicity and body mass index (Tepper *et al.* 2012).

Testosterone contributes to sexual desire in women (Wåhlin-Jacobsen *et al.* 2015), and research also indicates that it may play a role in cognition (Barrett-Connor & Goodman-Gruen 1999). Rosario *et al.* (2011) suggested that testosterone may have neuroprotective effects in men. However, corresponding studies in women are limited and not directly comparable (Dratva *et al.* 2024).

### MHT and cognition

Menopause hormone therapy (MHT) is used to manage menopausal symptoms (NICE 2024). In the UK and Europe, MHT includes oestrogen and progestogen treatment. The British Menopause Society (BMS) advises that testosterone be prescribed for low libido when there has been no improvement with systemic MHT and vaginal oestrogen. For this review, testosterone therapy was considered within the category of MHT, acknowledging that clinical regulatory frameworks vary across the world. As hormone levels fluctuate during different stages of menopause, the dose, delivery method and duration of MHT vary between individuals (Talaulikar 2022).

A recent systematic review of oestrogen-only and combined oestrogen–progestogen therapy (MHT) across 34 studies involving 14,914 women receiving MHT and 12,679 controls found no overall effect on cognition (Andy *et al.* 2024). However, further analysis suggested that oestrogen-only therapy may improve cognition in women following surgical menopause (Andy *et al.* 2024). This review also suggested that the timing of MHT initiation is important, with improvements observed when MHT is initiated in midlife or close to menopause onset rather than later in life, consistent with the ‘critical window hypothesis’.

Testosterone prescribing has anecdotally increased across NHS and private clinics in the UK, reflecting growing interest in its role during menopause. Women are increasingly asking questions and seeking support during this stage of life. Increased visibility of menopause in social and mainstream media may have helped reduce some elements of stigma associated with menopause more generally and contributed to interest in testosterone therapy. The quality of evidence and information shared through media outlets varies, meaning that some may be helpful while others may be misleading. Current BMS, NICE and IMS guidance only supports testosterone therapy for low libido (NICE 2024, BMS 2026, IMS 2026).

A previous Cochrane review by Somboonporn *et al.* (2005) explored whether adding testosterone to conventional postmenopausal hormone therapy was effective by comparing testosterone plus MHT with MHT alone. Although an important early contribution, this review treated cognition as only a secondary outcome. The authors did not evaluate testosterone therapy against a non-active control group, which may be important for detecting subtle cognitive effects, and did not explore differences between objective and subjective cognitive outcomes. More recently, Islam *et al.* (2019) reviewed the safety and efficacy of testosterone therapy in women, with cognitive performance as one of several primary outcomes. This review permitted active comparator trial arms and did not explore differences between objective and subjective cognition.

Given these limitations, a more focused and up-to-date review is warranted.

### Rationale and aims

This systematic review and meta-analysis aimed to explore the effect of testosterone therapy on cognition in women during MT and postmenopause.

The key research questions were as follows:

- Is testosterone therapy associated with objective or subjective changes in cognition in women during MT and postmenopause?
- Do testosterone treatment characteristics (including dose, formulation and delivery method), age at initiation or duration of use, affect cognitive outcomes?

## Method

### Search strategy and selection criteria

The review was registered on PROSPERO in February 2025 (registration number CRD420250644676) and followed the Preferred Reporting Items for Systematic Reviews and Meta-Analyses (PRISMA) guidelines (Page *et al.* 2021). Five databases were searched from inception to 25 March 2025: PsycINFO, MEDLINE, Embase, Web of Science and the Cochrane Library. As part of the search process, the reference lists of relevant review papers were also checked to identify any additional eligible studies.

Inclusion and exclusion criteria were prespecified and followed the Population, Intervention, Comparison, Outcome (PICO) framework. Searches were limited to peer-reviewed articles involving human participants and published in English. Grey literature was excluded. Full search terms are available in Supplementary Table 1 (see section on Supplementary materials given at the end of the article).

Studies were included if they i) were RCTs, non-randomised intervention studies or observational studies; ii) included women in MT, postmenopause or surgically or medically induced menopause. Menopause status was defined by each study’s criteria, such as confirmation of amenorrhoea/surgical procedure or serum hormone thresholds), iii) administered testosterone therapy alone or in combination with stable background oestrogen therapy across all arms; iv) reported details of testosterone therapy (dose, formulation and delivery method), participant age and intervention duration; v) included a non-active control or comparison group (e.g. waitlist control, standard care or placebo) to ensure that between-group differences reflected testosterone therapy and were not confounded by the effects of another active treatment; and vi) used a valid and reliable measure of objective or subjective cognition. Studies were excluded if they i) fell outside of MT or postmenopause; ii) included women with neurological disorders or neurodegenerative conditions affecting cognition; iii) used medications or psychotherapies targeting cognition (e.g. stimulant medication); iv) were review papers, background information papers or opinion pieces without primary data; or v) used an active hormonal comparison. Eligibility screening was performed in Rayyan (https://www.rayyan.ai) by the first reviewer (AF), with a second reviewer (SAE) cross-checking 10% of the results. Any discrepancies were resolved through discussion.

### Quality assessment

The CASP RCT and Cohort Checklist (Critical Appraisal Skills Programme 2023) was used to assess the overall methodological quality of each study. The findings were not used as a scorecard or exclusion criterion but as a guide to assessing each paper, as recommended by the Cochrane handbook (Higgins *et al.* 2024). See Tables 1 and 2.

**Table 1:** CASP randomised controlled trial checklist.

	Davis *et al.* (2014)	Huang *et al.* (2015)	Kocoska-Maras *et al.* (2011)	Sherwin (1988)
Did the study address a clearly formulated research question?	Yes	Yes	Yes	Yes
Was the assignment of participants to interventions randomised?	Yes	Yes	Yes	Yes
Were all participants who entered the study accounted for at its conclusion?	Yes	Yes	Yes	Yes
Were the participants ‘blind’ to intervention they were given?	Yes	Yes	Yes	Yes
Were the investigators ‘blind’ to the intervention they were giving to participants?	Yes	Yes	Yes	Yes
Were the people assessing/analysing outcome/s ‘blinded’?	Yes	Yes	Yes	Yes
Were the study groups similar at the start of the randomised controlled trial?	Yes	Yes	Yes	Yes
Apart from the experimental intervention, did each study group receive the same level of care (that is, were they treated equally)?	Yes	Yes	Yes	Yes
Were the effects of intervention reported comprehensively?	Yes	Yes	Yes	No
Was the precision of the estimate of the intervention or treatment effect reported?	Yes	Yes	Yes	No
Do the benefits of the experimental intervention outweigh the harms and costs?	Yes	No	No	Yes
Can the results be applied to your local population/in your context?	Yes	No	Yes	No
Would the experimental intervention provide greater value to the people in your care than any of the existing interventions?	Yes	No	No	Yes

**Table 2:** CASP cohort studies.

Checklist	Davison *et al.* (2011)
Are the results valid?	Yes
Was the cohort recruited in an acceptable way?	Yes
Were all being the exposure accurately measures to minimise bias?	Yes
Was the outcome accurately measured to minimise bias?	Yes
Have the authors identified all important cofounding factors?	Yes
Have they taken account of the confounding factors in the design and/or analysis?	Yes
Was the follow-up of subjects complete enough?	Yes
What are the results of this study?	Yes
How precise are the results?	Yes
Do you believe the results?	Yes
Can the results be applied to a local population?	Yes
Do the results of this study fit with other available evidence?	Yes
What are the practical implications of this study?	Yes

### Data extraction

Data were extracted using a standardised extraction template that included study characteristics (e.g. study design, country, menopause stage and menopause stage classification tool), intervention details (e.g. delivery method, dose and duration), comparison group and cognitive outcomes (e.g. objective and subjective measures), domain classification, post-intervention means (*M*), standard deviations (*SD*) and sample sizes (*n*).

Where reported, change scores (*M and SD*) were extracted. Where studies reported interquartile ranges (IQRs) instead of *SD*, *SD* was approximated using the formula *SD* ≈ IQR/1.35 (Wan *et al.* 2014). Where medians rather than means were reported, these were extracted without conversion. When data were incomplete, the authors were contacted to request the raw or supplementary data. Papers with missing data were excluded from the meta-analysis and reported narratively. Each data entry was double-checked by a second rater (RD), and any discrepancies were discussed.

### Meta-analysis

Cognitive outcomes reported across these studies were categorised into corresponding cognitive domains based on the information provided in the original papers, cross-checked against a reliable compendium (Strauss *et al.* 2006) and discussed with a neuropsychologist and a research fellow. Cognitive domains represented by a single study were excluded from the meta-analyses and analysed narratively.

This process resulted in three cognitive domains: i) executive function, ii) visuospatial learning and memory and iii) verbal learning and memory. When multiple tests were reported, one measure per domain per study was selected to avoid violating the assumption of independent data points. Predefined ‘decision rules’ were applied to determine which test to include within each domain (Higgins *et al.* 2024). Specifically, the most similar objective tests were selected for each domain, with similarity defined primarily by the specific cognitive area assessed and the testing procedures used (see Table 3 and Supplementary data). Where multiple testosterone doses were reported in Huang *et al.* (2015), one dose was selected to maximise comparability with doses used in other studies and reduce heterogeneity by avoiding further dose variation, consistent with an exploratory meta-analysis. A meta-analysis was conducted where two or more non-overlapping studies reported data that could be used to calculate an effect size for an outcome. Hedges’ *g* effect sizes were calculated using the Campbell Collaboration effect size calculator (Campbell Collaboration 2023). A random-effects model was used to account for between-study heterogeneity. Heterogeneity was assessed using *I^2^* and *Q* statistics.

**Table 3:** Summary of objective cognitive tests used in the meta-analysis.

Reference	Objective cognitive domain	Objective cognitive test	Description of test
Davis *et al.* (2014)	Executive function	GMLT	The GMLT is a computerised version of the HPML task paradigm, stepping stone mazes. In this task a pathway must be located and learnt over successive trials within a grid of tiles according to a set of simple searching rules. A lower score equals a better performance
Huang *et al.* (2015)	Executive function	Trail making B test	Participants are asked to connect numbers (1–13) and letters (A–L) alternately (1-A–2-B…) on a piece of paper as quickly as possible (Huang *et al.* 2015). Scoring is expressed in terms of the time in seconds required for completion of each task (Strauss *et al.* 2006). A lower score equals a better performance
Davis *et al.* (2014)	Verbal learning and memory	ISLT immediate	The ISLT is part of CogState, a computerised battery of cognitive tests. The ISLT is a 12 word, three trial word list learning test in which individuals are instructed to remember a list of items that are found commonly in their local stores. A higher score equals better performance
Huang *et al.* (2015)	Verbal learning and memory	BSRT immediate	Participants are read 12 words and asked to recall as many words as possible. Subsequent trials include only those words that were not recalled on the immediately preceding trial (Huang *et al.* 2015). A higher score equals better performance
Kocoska-Maras *et al.* (2011)	Verbal learning and memory	Noun recall	Noun recall is a test of episodic verbal memory. A list of 12 common unrelated nouns are presented verbally with the instruction to remember as many as possible for an immediate free recall test. The words should be presented at two second intervals. A higher score equals better performance
Davis *et al.* (2014)	Visuospatial learning and memory	Continuous paired associate learning	The continuous paired associate learning is also from part of the CogState, a computerised battery of cognitive tests. In this task participants must match abstract patterns to their locations on the screen. A higher score equals better performance
Huang *et al.* (2015)	Visuospatial learning and memory	VSLT immediate	The VSLT requires the participant to learn to recognise seven abstract designs and recall the correct placement of each design on 6 × 4 matrix grid. After being shown the grid and correct placement, designs are removed and participants place the designs in the correct location. There are five learning trials and a recall administration after a 30-min delay (Huang *et al.* 2015). A higher score equals better performance

GMLT, Groton Maze Learning Task; HPML, Hidden Pathway Maze Learning; ISLT, International Shopping List; VSLT, visual spatial learning test; BSRT, Buschke Selective Reminding Test.

Analyses were conducted in R (v4.5.2) using the metafor package (v4.8-0) (Viechtbauer 2010).

### Narrative review

A narrative review was conducted when studies were not eligible for quantitative pooling, or when only a single study contributed data to a cognitive domain, making meta-analysis impossible. In addition, when studies contributed multiple tests within the same cognitive domain, only one outcome was included in the meta-analysis, as described above. The remaining outcomes were reported narratively.

Testosterone doses were standardised to 1 mg/day for comparability across studies, following consultation with an endocrinologist (NK), with the acknowledgement that this does not represent biological equivalence but allows for descriptive comparison.

## Results

In total, 3,880 studies were identified through database searches (see Fig. 1). After removal of duplicates (*n* = 1,014), 2,866 studies were screened by title and abstract, and 56 full texts were assessed. A total of five papers were eligible for inclusion, of which three were eligible for inclusion in the meta-analyses. No additional studies were identified through backward reference sampling of relevant reviews. No studies were identified that examined subjective cognition in women during MT. All included studies involved postmenopausal women. Study characteristics are summarised in Table 4.

**Figure 1:**
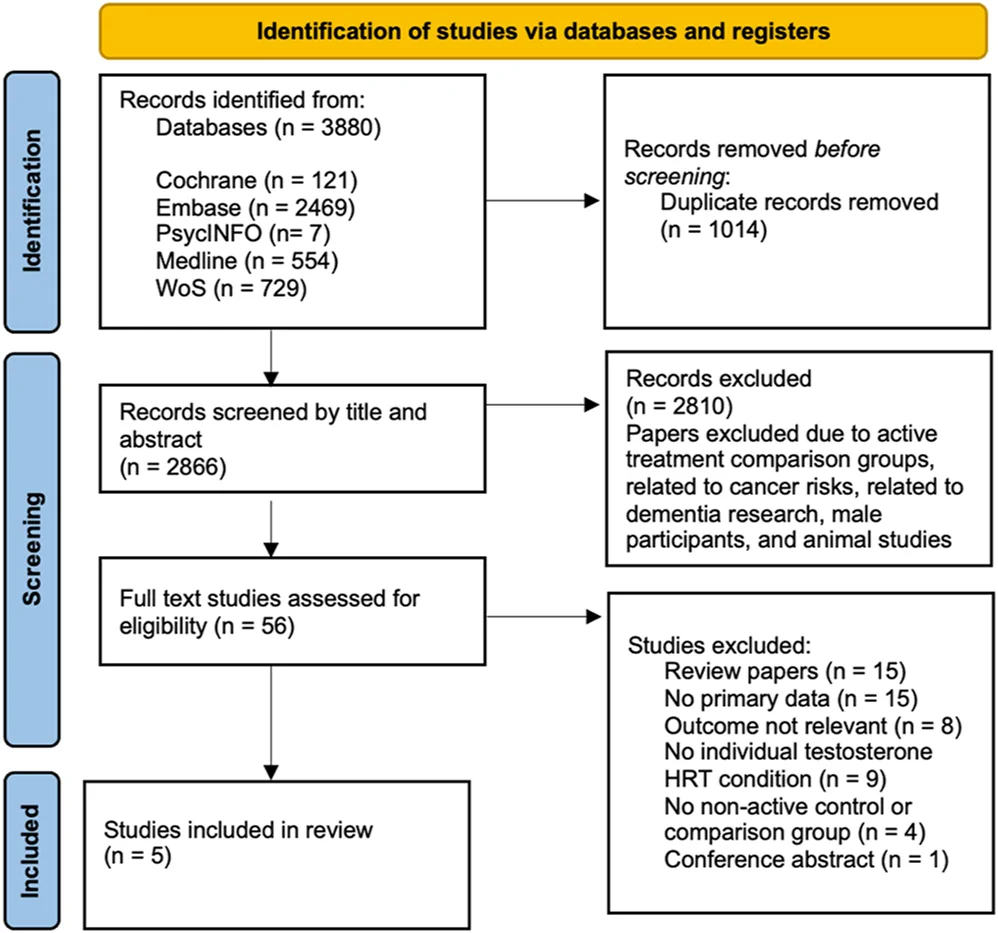
PRISMA 2020 flow diagram of the study selection process.

**Table 4:** Characteristics of included studies.

Characteristics	Davis *et al.* (2014)	Davison *et al.* (2011)	Huang *et al.* (2015)	Kocoska-Maras *et al.* (2011)	Sherwin (1988)
Country	Australia	Australia	USA	Sweden	Canada
Design	RCT	Non-randomised	RCT	RCT	RCT-crossover
Menopause stage	Postmenopausal	Postmenopausal	Postmenopausal	Postmenopausal	Postmenopausal
Menopause classification	Atleast 12 months of amenorrhoea	55 years or greater or bilateral oophorectomy or >12 months amenorrhoea and FSH > 20 IU/L, or treated for climacteric symptoms with postmenopausal oestrogen therapy for >12 months	Hysterectomy alone or partial oophorectomy if their FSH levels were ≥30 U/l or if they were already receiving oestrogen therapy	At least 12 months of amenorrhoea or serum levels of FSH >30 U/L	Total abdominal hysterectomy + bilateral salpingo-oophorectomy
Recruitment age (years)	55–65	45–60	41–62	50–62	N/A
Average age (years)	Median: 60	Mean: 57.2	Mean: 54	Range: 50–65; T: 58.3 (mean); C: 5,801 (mean)	45.4
Intervention	Testosterone therapy for naturally menopausal women	Testosterone therapy for natural and surgical menopausal women	Testosterone therapy for women who had undergone hysterectomy with or without partial or total oophorectomy	Testosterone therapy for naturally menopausal women	Testosterone therapy for surgical menopause
Stabilising period pretrial	None	Stable oestrogen therapy for ≥8 weeks prior to testosterone	Transdermal oestrogen for 12 weeks before randomisation	None reported	None reported
Testosterone delivery method	Transdermal gel	Transdermal spray	Intramuscular injection	Oral	Injection
Testosterone formulation	LibiGel	Unreported	Enanthate	Undecanoate	Enanthate
Testosterone dose	0.3 mg/day	4.55 mg/day delivered (0.3 mg/day absorbed)	25 mg*/week	40 mg/day	200 mg/28 days
Treatment duration	26 weeks	26 weeks	24 weeks	4 weeks	2 × 3 months with 1 month placebo washout
Comparator	Placebo gel	Treatment as usual control	Placebo injection	Placebo tablet	Placebo injection
Total sample size	89	39	21*	134	20

RCT, randomised controlled trial; T, testosterone therapy; C, comparator; N/A, data not available.

* Individual dose and corresponding sample size selected for meta-analysis.

### Objective cognition

Three studies that included measures of objective cognition were eligible for inclusion in the meta-analysis, all of which sampled postmenopausal women. Two studies were not eligible for meta-analysis but were eligible for narrative review. Publication bias could not be meaningfully assessed because there were fewer than ten studies in each analysis and funnel plots could not be reliably evaluated.

A separate random-effects meta-analysis was conducted for each of the three predefined cognitive domains, as defined *a priori*. Forest plots were used to graphically represent the meta-analysis. Forest plots showing standardised mean differences (SMD) of Hedges’ *g* and 95% confidence intervals are presented in Figs 2 and 3.

**Figure 2:**
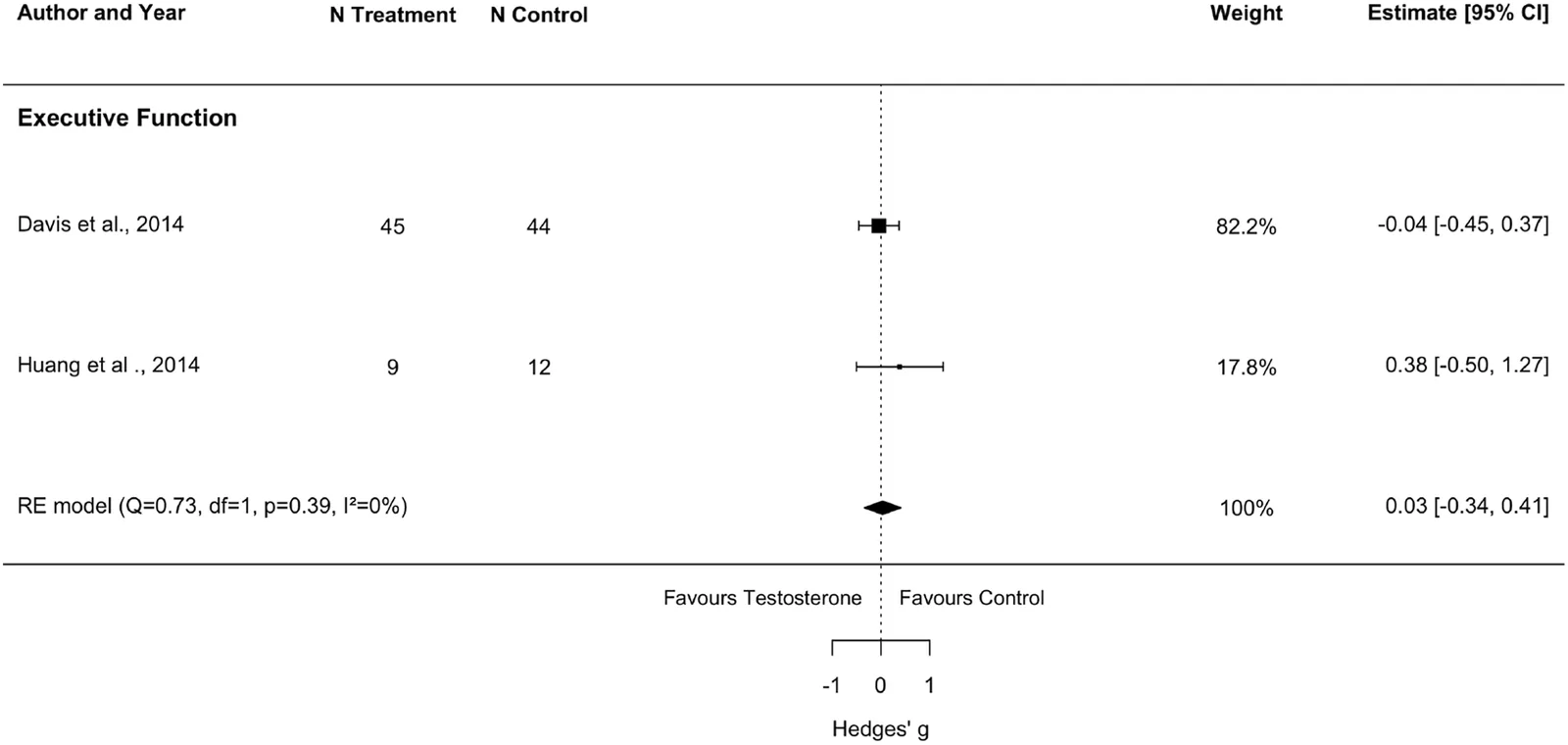
Forest plot for the cognitive domain of executive function.

**Figure 3:**
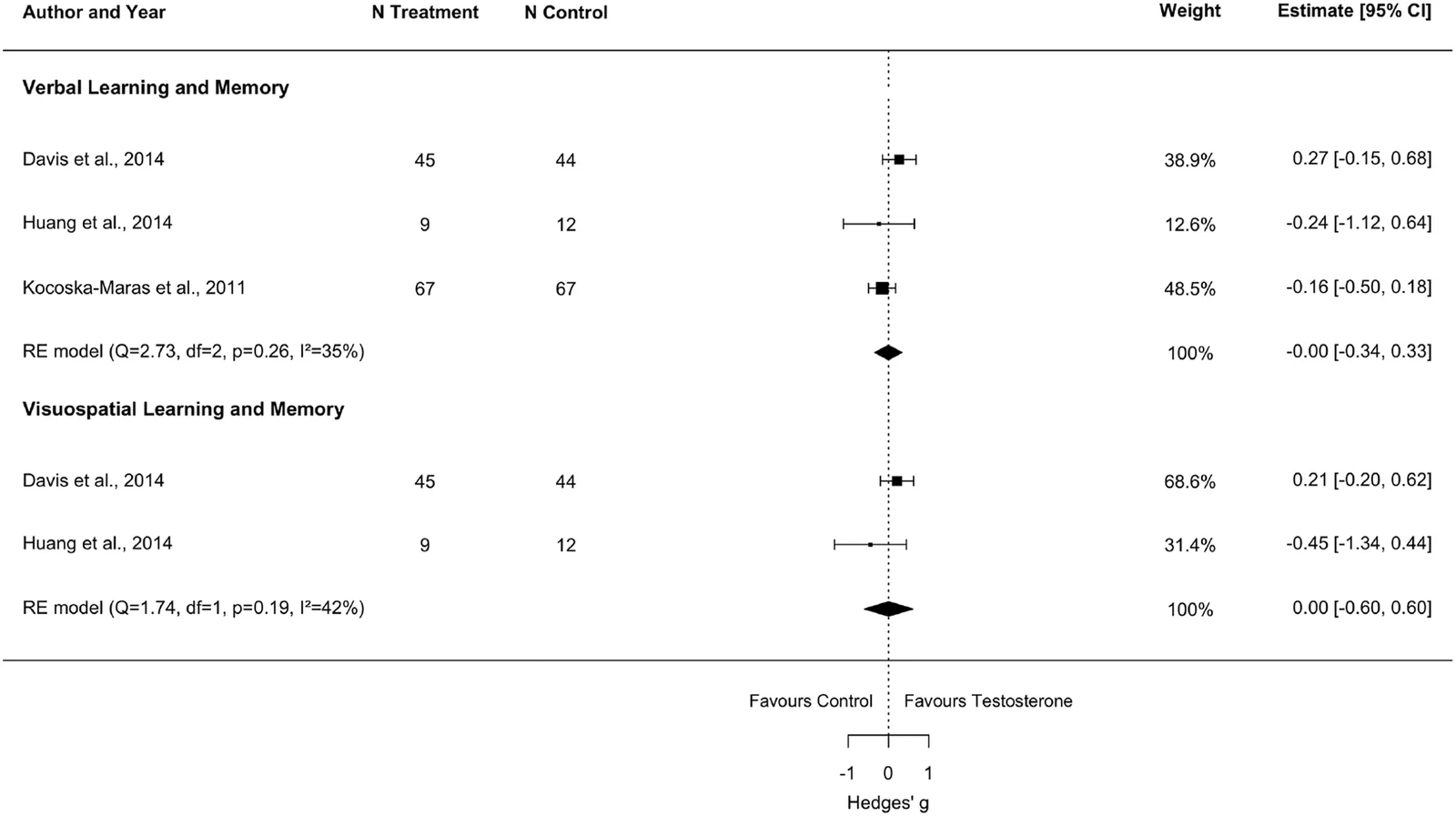
Forest plots for the cognitive domains of verbal learning and memory and visuospatial learning and memory.

Across all three cognitive domains, no statistically significant effects of testosterone on cognitive outcomes were observed in postmenopausal women. Heterogeneity varied across the cognitive domains, reflecting between-study differences in intervention and participant characteristics.

#### Executive function

A meta-analysis of two studies (Davis *et al.* 2014, Huang *et al.* 2015) (*n* = 110) found a pooled Hedges’ *g* of 0.03 (95% CI: −0.34 to 0.41), indicating no statistically significant pooled effect. Heterogeneity was low, with no significant variation between the samples (*Q* = 0.73, *I^2^* = 0%, *P* = 0.39).

#### Verbal learning and memory

A meta-analysis of three studies (Kocoska-Maras *et al.* 2011, Davis *et al.* 2014, Huang *et al.* 2015) (*n* = 244) found a pooled Hedges’ *g* of −0.00 (95% CI: −0.34 to 0.33), indicating no statistically significant pooled effect. Heterogeneity was moderate (*Q* = 2.73, *I^2^* = 35%, *P* = 0.26).

#### Visuospatial learning and memory

A meta-analysis of two studies (Davis *et al.* 2014, Huang *et al.* 2015) (*n* = 110) found a pooled Hedges’ *g* of 0.00 (95% CI: −0.60 to 0.60), indicating no statistically significant pooled effect. Heterogeneity was moderate (*Q* = 1.74, *I^2^* = 42%, *P* = 0.19).

GRADE certainty assessment rated the evidence for all outcomes as very low due to risk of bias, inconsistency, indirectness and imprecision. See Table 5 for the certainty assessment.

**Table 5:** GRADE certainty of evidence.

Outcome	Number of		Effect estimate* (95% CI)	Certainty assessment	Comments	Certainty of evidence
	Studies	Participants				
Executive function	2	110	0.03 (−0.34, 0.41)	Serious risk of bias	Small samples limiting power to detect effects. Some limited information on randomisation procedures in some papers. Different populations (surgical menopause vs natural menopause), different testosterone interventions. Wide confidence interval spanning both sides of the null with different clinical implications	Very low
				No serious inconsistency		
				Serious indirectness		
				Serious imprecision		
				Cannot assess publication bias		
Verbal learning and memory	3	244	0.00 (−0.34, 0.33)	Serious risk of bias	Small samples limiting power to detect effects. Some limited information on randomisation procedures in some papers. Moderate heterogeneity and variation in effect size. Variation in menopause populations (surgical vs natural), variation in testosterone treatment. Wide confidence interval spanning both sides of the null with different clinical implications	Very low
				Serious inconsistency		
				Serious indirectness		
				Serious imprecision		
				Cannot assess publication bias		
Visuospatial learning and memory	2	110	0.00 (−0.60, 0.60)	Serious risk of bias	Small samples limiting power to detect effects. Some limited information on randomisation procedures in some papers. Moderate heterogeneity and variation in effect size. Variation in menopause populations (surgical vs natural), variation in testosterone treatment. Wide confidence interval spanning both sides of the null with different clinical implications	Very low
				Serious inconsistency		
				Serious indirectness		
				Serious imprecision		
				Cannot assess publication bias		

* Values are Hedges’ *g* (95% CI).

### Narrative review

Papers that could not be included in the meta-analysis were analysed narratively. Tests included in the meta-analysis were not reported narratively again. Sherwin (1988) was excluded due to the absence of extractable summary statistics and reported separately due to substantial methodological and measurement differences. Davison *et al.* (2011) was also excluded from the meta-analysis due to its non-randomised design, which did not meet the eligibility criteria for meta-analysis. In cognitive domains where only a single study contributed data, meta-analysis was not possible and findings were therefore reported narratively (Kocoska-Maras *et al.* 2011, Davis *et al.* 2014, Huang *et al.* 2015). Huang *et al.* (2015) reported multiple testosterone doses. The 25 mg arm was used for the meta-analysis, while the remaining doses (3, 6.25 and 12.5 mg) were included in the narrative review.

#### Attention and processing speed

Three studies reported outcomes in this domain. Davison *et al.* (2011) assessed this domain using the CogState detection task. They found no change in scores from baseline to week 26 in the testosterone arm and no difference compared with the normative control group. Bootstrapped regression, adjusted for age and baseline score, confirmed the absence of a treatment effect (*β* = 0, 95% CI: (−0.03, 0.04), *P* = 0.872). Davis *et al.* (2014) used the same CogState detection task and also reported no significant between-group difference in change from baseline to week 26 after adjusted for age and baseline scores (*β* = −0.01, 95% CI: (−0.02, 0.01), *P* = 0.38).

#### Executive function

Huang *et al.* (2015) additionally assessed executive function using the Stroop Colour Word Interference Test and Mazes. No significant changes (all *P* > 0.05) were observed in any testosterone treatment arm compared with placebo, even after adjustment for baseline cognitive function, age, and education. Davison *et al.* (2011) found no significant improvements in the Groton Maze Learning Task after 26 weeks of treatment. Regression analysis adjusted for age and baseline performance confirmed the absence of treatment effects (*β* = −4.24, 95% CI: (−13.04, 3.60), *P* = 0.308).

#### Language and verbal fluency

Only Huang *et al.* (2015) reported tests in this domain, using the Category Fluency and Phonemic Fluency. There were no significant differences observed between any testosterone treatment arms and the placebo group (all *P* > 0.05), even after adjustment for age, education and baseline cognitive function.

#### Spatial cognition

Kocoska-Maras *et al.* (2011) assessed spatial cognition using the Mental Rotation Task. They reported no significant differences between the testosterone treatment arm and the placebo group after 4 weeks, even after adjustment for age (*mean difference*: 0.34, 95% CI: (−1.48, 2.17), *P* > 0.05).

#### Verbal learning and memory

Huang *et al.* (2015) additionally assessed verbal learning and memory using Paragraph Recall (immediate and delayed) and the Buschke Selective Reminding Test. No significant changes were observed in any testosterone treatment arm compared with placebo, even after adjustment for baseline cognitive function, age and education (all *P* > 0.05). Davis *et al.* (2014) used the International Shopping List Test (delayed recall condition) and reported no statistically significant effects between the testosterone arm and the normative control arm after 26 weeks, following adjustment for age and baseline scores (*β* = −0.11, 95% CI: (−0.84, 0.61), *P* = 0.77). Davison *et al.* (2011) also reported scores for the International Shopping List Test (immediate and delayed recall conditions). In the delayed recall condition, week 26 scores were significantly higher in the testosterone arm than in the normative control group (*P* < 0.05). Regression analyses adjusted for age and baseline performance confirmed significant treatment effects for both immediate and delayed recall conditions (immediate: *β* = 2.03, 95% CI: (0.49, 3.43), *P* = 0.007; delayed recall: *β* = 1.27, 95% CI: (0.11, 2.18), *P* = 0.015).

#### Visuospatial learning and memory

Huang *et al.* (2015) additionally assessed visuospatial learning and memory using the Puget Sound Route Learning Test (immediate and delayed) and the Visual Spatial Learning Test (delayed). No significant changes were observed in any testosterone treatment arm compared with placebo, even after adjustment for baseline cognitive function, age and education (all *P* > 0.05). Davison *et al.* (2011) reported improvements from baseline to 26 weeks in the Groton Maze (recall) in the testosterone arm (*P* < 0.05), although a regression analysis adjusted for age and baseline performance did not show a significant effect (*β* = −1.49, 95% CI: (−2.95, 0.40), *P* = 0.077). Davis *et al.* (2014) also reported no significant difference between the testosterone arm and placebo on the Groton Maze (recall). Davis *et al.* (2014) reported no significant difference between the testosterone treatment arm and placebo on the ‘Groton maze task recall’ (*β* = −0.11, 95% CI: (−0.84, 0.61), *P* > 0.77).

#### Working memory

Davison *et al.* (2011) assessed working memory using the CogState One-Back Task. They reported no improvement from baseline to week 26 in the testosterone arm, and no difference compared with the normative control group. Regression analysis adjusted for age and baseline performance confirmed no significant effect (*β* = 0.01, 95% CI: (−0.03, 0.06), *P* = 0.700). Huang *et al.* (2015) used Letter-Number Sequencing and reported no significant changes in any testosterone treatment arm compared with placebo, even after adjustment for baseline cognitive function, age and education (all *P* > 0.05).

Sherwin (1988) was included in this narrative review but reported separately due to substantial methodological and measurement differences. The authors investigated the effects of androgen replacement therapy (as well as oestrogen-only and combined oestrogen−androgen therapy) in surgically menopausal women with a mean age of 45.4 years, using a randomised crossover design. Each treatment phase was separated by a one-month placebo washout period to reduce possible carryover effects. Objective cognitive performance was assessed using measures of short- and long-term memory, including i) digit span subtest as a measure of short-term memory, ii) clerical speed and accuracy as measures of response speed, iii) paragraph recall as a measure of memory and iv) abstract reasoning as a measure of reasoning ability. This paper had limited statistical reporting; however, the overall results indicated improvements in cognitive performance in the testosterone intervention group (and other treatment groups) compared with the placebo group.

### Subjective cognition

No studies were identified that measured subjective cognition and met the inclusion criteria.

### Testosterone characteristics, age and intervention duration

Among the three studies included in the meta-analysis, testosterone characteristics (route of administration, formulation and dose), age, and intervention duration varied substantially. Variability was also observed in studies described narratively. This, along with the use of small datasets, limited the direct comparability of these studies and prevented further meta-analyses. A narrative approach was adopted to describe characteristics that may contribute to variability between studies in objective cognitive outcomes.

#### Testosterone characteristics

##### Delivery method

Delivery methods varied considerably. Two studies used transdermal administration (Davison *et al.* 2011, Davis *et al.* 2014), one used oral administration (Kocoska-Maras *et al.* 2011) and two used intramuscular injections (Sherwin 1988, Huang *et al.* 2015). These delivery methods are associated with different pharmacokinetics, including how quickly the exogenous hormone enters the bloodstream, reaches peak concentration and thereafter the rate of decay of the plasma hormone level.

##### Formulation

Multiple formulations were used. Two studies (Davison *et al.* 2011, Davis *et al.* 2014) reported using a testosterone preparation but did not specify the formulation, compared with testosterone undecanoate reported by Kocoska-Maras *et al.* (2011) and testosterone enanthate by Huang *et al.* (2015) and Sherwin (1988).

##### Dose

Testosterone doses were standardised to 1 mg/day for comparability across studies, following consultation with an endocrinologist, with the acknowledgement that this does not accurately reflect the overall impact of delivery method, formulation, and dose on the body’s metabolism of each testosterone preparation. It does allow for some descriptive comparison. Across the studies, there was variation, ranging from 0.3 mg/day (Davis *et al.* 2014), 4.55 mg/day delivered with 0.3 mg/day absorbed (Davison *et al.* 2011), 25 mg/week (≈3.6 mg/day) (Huang *et al.* 2015), 40 mg/day (Kocoska-Maras *et al.* 2011) to 200 mg/month (≈6.7 mg/day) (Sherwin 1988).

#### Age

Across the five studies, participants’ ages ranged from 41 to 65 years, with an approximate mean age of 57 years, consistent with the reporting of all studies that recruited postmenopausal women (Huang *et al.* 2015). recruited the youngest cohort (*M* = 54 years) compared to (Davison *et al.* 2011, Kocoska-Maras *et al.* 2011, Davis *et al.* 2014), who reported slightly older cohorts in their mid-to-late 50s. Sherwin (1988) do not clearly report participant ages. There were no eligible studies in MT that met the inclusion criteria.

#### Duration

Across these studies, intervention duration ranged from four weeks (Kocoska-Maras *et al.* 2011) to 24–26 weeks (Davison *et al.* 2011, Davis *et al.* 2014, Huang *et al.* 2015). For Sherwin (1988), a crossover design meant that participants experienced two three-month treatment periods with a washout period in between.

## Discussion

### Summary of main findings

Across three studies of postmenopausal women spanning three cognitive domains, meta-analyses indicated no statistically significant effects, with effect estimates close to zero. Heterogeneity varied across domains, suggesting inconsistency between study findings. There was substantial variability in testosterone characteristics, intervention duration and participant age. No studies were identified that used a population in MT or assessed subjective cognition. In summary, the current review is most likely underpowered (*k* = 2–3; *n* = 244) and is insufficient to conclude that there is no effect, rather than providing evidence of no effect.

### Interpretation

Given the inconclusive findings, comparisons with the broader literature are limited. Some research suggests improved spatial ability when low testosterone levels are restored (Miller *et al.* 2005), whereas other research do not (Dong *et al.* 2021). Furthermore, some research suggests that higher levels of endogenous testosterone and oestrogen can benefit verbal memory. The literature presents mixed findings on the relationship between testosterone and cognition, and this relationship is rarely reported in midlife women, with findings often relying on extrapolated results.

Evidence from Greendale *et al.* (2009) suggests that cognitive difficulties during menopause may be time-limited. In their longitudinal cohort, postmenopausal women who were not using hormone therapy showed a return to premenopausal-like cognitive performance levels. Similarly, Maki (2015) reported a return to baseline memory state during the postmenopausal period. This is relevant to the current review, as all included studies sampled were already postmenopausal, a stage at which cognitive performance may have begun to recover, as suggested by Greendale *et al.* (2009) and (Maki 2015). If cognitive difficulties are restricted to MT, sampling women after this period reduces the chances of detecting treatment-related effects.

Another complementary idea is the ‘critical window hypothesis’ of hormone therapy and cognitive function, which states that the effects of hormone therapy depend on the timing of initiation with respect to age or MT, with optimal effects observed with early initiation (Maki 2013). This hypothesis explores treatment responsiveness rather than natural cognitive trajectories. Although the evidence for this hypothesis centres mainly on oestrogen rather than testosterone treatment, it could indicate that initiating testosterone treatment earlier in menopause may increase the likelihood of influencing cognitive outcomes.

Substantial differences in the number of studies measuring objective versus subjective cognition in this review could be understood within a biopsychosocial framework. This framework proposes that hormones interact with psychological factors (e.g. beliefs and symptom attributions), as well as social and contextual factors, to shape cognitive experiences. As a result, subjective experiences may not correlate with objective performance. From this perspective, objective neuropsychological testing measures may lack sensitivity to measure subjective cognitive complaints. Furthermore, this may indicate a lack of appropriate tools to assess subjective cognition in menopause, which could explain why no studies were identified in our search.

### Strengths and limitations

This is an up-to-date and focused systematic review and meta-analysis exploring the effects of testosterone on objective and subjective cognition in women in MT and postmenopause. This study was not designed to examine the mechanisms by which testosterone impacts cognitive function, but aimed to explore trends in the data.

A strength of this review is the inclusion of both surgical and natural menopause, increasing the generalisability of the findings. This inclusion also resulted in methodological diversity, as two of the five studies employed an oestrogen stabilisation period of at least 8 weeks. This reflects real-world clinical variation among women.

Another strength of this review is the use of a screening process to ensure that reliable and valid cognitive measures were included. Predefined decision rules were used to select a single test per cognitive domain for studies that reported multiple tests. This test selection was intended to reduce violations of statistical independence.

Limitations of this review include methodological constraints. Small sample sizes and differences in study designs and comparator groups may affect the validity and comparability of this review. Previous similar reviews were also checked to ensure that relevant trials were not missed. Of the five trials reported in Somboonporn *et al.* (2005), four did not meet the eligibility criteria for this review because they used active hormonal comparators, and one study (Sherwin 1988) was eligible but could only be analysed narratively because extractable data were unavailable. Of the three cognition trials reported in Islam *et al.* (2019), two were excluded for the same eligibility reasons, and one study (Davis *et al.* (2014) was included.

A further limitation is that the objective test selection process (selecting a single test per cognitive domain for studies that reported multiple measures) may have introduced variation in test sensitivity. The selected test’s ability to detect cognitive domain-specific changes may have been influenced both positively and negatively. Furthermore, neuropsychological tests may represent more than one cognitive domain.

Another limitation of this review was the lack of studies exploring subjective cognition. One study was excluded during the screening stages of this review. Glynne *et al.* (2025) conducted a retrospective cohort study of 510 perimenopausal and postmenopausal women using MHT, assessing self-reported mood and cognitive symptoms. However, without a comparison or control group, the reliability of these findings is limited, and the reported outcomes could be attributable to expectancy or placebo effects.

Variations in testosterone characteristics were also a limiting factor in this review. There were notable differences in the delivery methods and formulations of testosterone. These variations can affect absorption, drug stability and metabolic processes. Therefore, differences in cognitive outcomes may not be fully attributable to testosterone therapy alone, given its pharmacokinetic properties. The duration of testosterone therapy ranged from 4 weeks to 24–26 weeks (approximately 6 months). Testosterone may take 3–6 months to achieve full dose effects (Panay 2022), which raises the question of whether a 4-week treatment duration would be sufficient to detect cognitive changes. No studies explored long-term follow-up, limiting any conclusions about the persistence or decline of effects over time.

The ages of participants across the included studies ranged from 41 to 65 years (mean average ≈ 57 years), spanning MT to postmenopause (Santoro 2016, McCarthy & Raval 2020). Although all participants in this review were confirmed to be postmenopausal, no standardised categorisation was used (e.g., STRAW+10; Harlow *et al.* 2012), nor was the time since menopause at the start of the intervention recorded. It is also important to consider that any classification may mask the effects of biological ageing. Cognitive outcomes in midlife are thought to be influenced by both chronological ageing (e.g. structural and functional changes in the nervous system (Salthouse 2010)) and hormonal status. Therefore, age may be a confounding variable, limiting the ability to attribute cognitive changes to testosterone therapy.

Finally, there are limitations to comparing surgical menopause (i.e. a sudden and total switch off in hormones) with physiological menopause (i.e. a gradual decline in hormone levels). Furthermore, if a bilateral oophorectomy was performed for reasons of malignancy, other factors, such as medication (e.g. chemotherapy) or psychological trauma, may also impact cognition.

### Implications for future research and practice

Based on this review, it remains unclear whether testosterone improves cognition in MT and postmenopausal women, owing to the low statistical power analysis and limited number of included studies. Many factors that require consideration in further research before conclusions regarding the effectiveness of testosterone therapy on cognition can be drawn.

Future trials should include a non-active comparison group and clearly detail the drugs’ characteristics (e.g. dose, formulation, and delivery method) with clear rationales for their selection. This would allow the pharmacokinetic properties of testosterone to be carefully considered. Furthermore, there is a need to explore testosterone therapy in MT. Cognitive complaints appear to peak during the menopause transition (Unkenstein *et al.* 2016) when hormone fluctuations are greatest. Early discussions from Greendale *et al.* (2009) and ideas alongside the critical window hypothesis of hormone therapy and cognitive function suggest the importance of exploring testosterone therapy initiation during MT.

Differences in findings between objective and subjective cognition may reflect underlying epistemological approaches. Objective cognition is often assessed within a biomedical framework, whereas subjective cognition aligns more closely with a phenomenological epistemology, emphasising lived experience. An integrative approach combining medicine and psychology may be required to explore the possible effects of testosterone on subjective cognition.

This review reports underpowered and insufficient evidence to prescribe testosterone therapy for cognitive benefit, raising considerations for clinical practice. Although off-label and individualised care can be considered at times in menopause symptom management, women must understand the current lack of robust evidence for testosterone therapy and cognition. This review does highlight the continued need for a biopsychosocial approach to the management of cognitive complaints and integration between medical prescribing and psychological support.

## Conclusion

This review concludes that the evidence is underpowered and insufficient, rather than evidence of no effect, to explore the effect of testosterone therapy on cognition for women in MT and postmenopause. Cognitive difficulties during MT and postmenopause can impact quality of life, including reduced self-confidence, difficulties at work, and anxiety. Evidence for other forms of MHT remains mixed, and the role of testosterone for objective or subjective cognitive complaints remains underexplored and further research is required.

## Supplementary materials



## Declaration of interest

There is no conflict of interest that could be perceived as prejudicing the impartiality of the research reported.

## Funding

This research did not receive any specific grant from any funding agency in the public, commercial or not-for-profit sector.

## Author contribution statement

AF, AS and RD participated in the conception and design of the study. AF conducted the searches and data extraction and performed the statistical analyses. SAE conducted a secondary review of screening papers. RD conducted secondary reviews of all data extraction and analyses. NK provided medical supervision during the interpretation of study outcomes and implications. AS and RD provided supervision across all stages of the study. All authors approved the final version of the manuscript.
