# Supplementary material for: Testosterone therapy for cognition in menopause: A systematic review and meta-analysis
Source: Reprod Fertil. 2026 Oct 30;7(4):RAF260080. doi: 10.1530/RAF-26-0080 (PMC13745893; doi:10.1530/RAF-26-0080)
Supplement: Supplementary file 1 [file RAF-26-0080_supplementary_table_1.pdf]

Table 1 Search term details for each database.

PsycINFO	<1806 to March 2025 Week 3>
	1 exp Menopause/
	2 (Menopaus* or peri?menopaus* or peri-menopaus* or post?menopaus* or post-menopaus*).ab,id,ti.
	3 ((end or cessation or post) adj2 (menstruation or menses or "menstrual period" or "reproductive age")).ab,id,ti
	4 1 or 2 or 3
	5 exp Testosterone/
	6 exp Androgens/
	7 ("hormone replacement therap*" or HRT or testosterone or androgen*).ab,kw,ti.
	8 5 or 6 or 7
	9 exp Cognition/
	10 "cognit*".ab,kw,ti.
	11 9 or 10
	12 4 and 8 and 11 7
MEDLINE	<1946 to March 26, 2025>
	1 exp Menopause/
	2 (Menopaus* or peri?menopaus* or peri-menopaus* or post?menopaus* or post-menopaus*).ab,kw,ti.
	3 ((end or cessation or post) adj2 (menstruation or menses or "menstrual period" or "reproductive age")).ab,kw,ti.
	4 1 or 2 or 3
	5 exp Testosterone/
	6 exp Androgens/
	7 ("hormone replacement therap*" or HRT or testosterone or androgen*).ab,kw,ti.
	8 5 or 6 or 7
	9 exp Cognition/
	10 "cognit*".ab,kw,ti.
	11 9 or 10 696280
	12 4 and 8 and 11
Embase	<1974 to 2025 March 26>
	1 exp menopause/
	2 (Menopaus* or peri?menopaus* or peri-menopaus* or post?menopaus* or post-menopaus*).ab,kw,ti.
	3 ((end or cessation or post) adj2 (menstruation or menses or "menstrual period" or "reproductive age")).ab,kw,ti.
	4 1 or 2 or 3
	5 exp testosterone/
	6 exp androgen/ 2
	7 ("hormone replacement therap*" or HRT or testosterone or androgen*).ab,kw,ti.
	8 5 or 6 or 7
	9 exp cognition/
	10 Cognit*.ab,kw,ti.
	11 9 or 10 3631307
	12 4 and 8 and 11
Web of Science	TS=(Menopaus* or peri?menopaus* or peri-menopaus* or post?menopaus* or post-menopaus*) OR TI=((end or cessation or post) NEAR/2 (menstruation or menses or "menstrual period" or "reproductive age")) OR AB=((end or cessation or post) NEAR/2 (menstruation or menses or "menstrual period" or "reproductive age"))
	testosterone or androgens or "hormone replacement therap*" or
	HRT (Topic)
	cognit* (Topic)
Cochrane Library	#1 MeSH descriptor: [Menopause] explode all trees
	#2 (Menopaus* or peri?menopaus* or peri-menopaus* or post?menopaus* or post-menopaus*):ti,ab,kw
	#3 ((end or cessation or post) NEAR/2 (menstruation or menses or "menstrual period" or "reproductive age")):ti,ab,kw
	#4 MeSH descriptor: [Testosterone] explode all trees
	#5 MeSH descriptor: [Androgens] explode all trees
	#6 (testosterone or androgen* or hormone replacement therap* or HRT):ti,ab,kw
	#7 MeSH descriptor: [Cognition] explode all trees
	#8 (cognit*):ti,ab,kw
	#9 searches #1 or #2 or #3 and #4 or #5 or #6 and #7 and #8
